# Targeting the metabolic vulnerability of acute myeloid leukemia blasts with a combination of venetoclax and 8-chloro-adenosine

**DOI:** 10.1186/s13045-021-01076-4

**Published:** 2021-04-26

**Authors:** Ralf Buettner, Le Xuan Truong Nguyen, Corey Morales, Min-Hsuan Chen, Xiwei Wu, Lisa S. Chen, Dinh Hoa Hoang, Servando Hernandez Vargas, Vinod Pullarkat, Varsha Gandhi, Guido Marcucci, Steven T. Rosen

**Affiliations:** 1 Hematology Malignancies Research Institute, Gehr Family Center for Leukemia Research, City of Hope Medical Center, Kaplan CRB, 1026, 1500 East Duarte Road, Duarte, CA 91010 USA; 2 Integrative Genomics Core, Beckman Research Institute, City of Hope Medical Center, Duarte, CA USA; 3 Department of Experimental Therapeutics, The University of Texas MD Anderson Cancer Center, Houston, TX USA

**Keywords:** Acute myeloid leukemia, Oxidative phosphorylation, Fatty acid oxidation, Nucleoside analog, Metabolism

## Abstract

**Background:**

BCL‐2 inhibition through venetoclax (VEN) targets acute myeloid leukemia (AML) blast cells and leukemic stem cells (LSCs). Although VEN-containing regimens yield 60–70% clinical response rates, the vast majority of patients inevitably suffer disease relapse, likely because of the persistence of drug-resistant LSCs. We previously reported preclinical activity of the ribonucleoside analog 8-chloro-adenosine (8-Cl-Ado) against AML blast cells and LSCs. Moreover, our ongoing phase I clinical trial of 8-Cl-Ado in patients with refractory/relapsed AML demonstrates encouraging clinical benefit. Of note, LSCs uniquely depend on amino acid-driven and/or fatty acid oxidation (FAO)-driven oxidative phosphorylation (OXPHOS) for survival. VEN inhibits OXPHOS in LSCs, which eventually may escape the antileukemic activity of this drug. FAO is activated in LSCs isolated from patients with relapsed AML.

**Methods:**

Using AML cell lines and LSC-enriched blast cells from pre-treatment AML patients, we evaluated the effects of 8-Cl-Ado, VEN and the 8-Cl-Ado/VEN combination on fatty acid metabolism, glycolysis and OXPHOS using liquid scintillation counting, a Seahorse XF Analyzer and gene set enrichment analysis (GSEA). Western blotting was used to validate results from GSEA. HPLC was used to measure intracellular accumulation of 8-Cl-ATP, the cytotoxic metabolite of 8-Cl-Ado. To quantify drug synergy, we created combination index plots using CompuSyn software. The log-rank Kaplan–Meier survival test was used to compare the survival distributions of the different treatment groups in a xenograft mouse model of AML.

**Results:**

We here report that VEN and 8-Cl-Ado synergistically inhibited in vitro growth of AML cells. Furthermore, immunodeficient mice engrafted with MV4-11-Luc AML cells and treated with the combination of VEN plus 8-Cl-Ado had a significantly longer survival than mice treated with either drugs alone (*p* ≤ 0.006). We show here that 8-Cl-Ado in the LSC-enriched population suppressed FAO by downregulating gene expression of proteins involved in this pathway and significantly inhibited the oxygen consumption rate (OCR), an indicator of OXPHOS. By combining 8-Cl-Ado with VEN, we observed complete inhibition of OCR, suggesting this drug combination cooperates in targeting OXPHOS and the metabolic homeostasis of AML cells.

**Conclusion:**

Taken together, the results suggest that 8-Cl-Ado enhances the antileukemic activity of VEN and that this combination represents a promising therapeutic regimen for treatment of AML.

**Supplementary Information:**

The online version contains supplementary material available at 10.1186/s13045-021-01076-4.

## Background

Acute myeloid leukemia (AML) is a genetically heterogeneous hematopoietic malignancy affecting all age groups, but particularly older individuals. In 2021, 20,240 new cases and 11,400 deaths are predicted to occur in the USA [1]. Conventional chemotherapy followed by allogeneic stem cell transplantation (alloSCT) may provide the best chance for long-term survival, but this intensive approach carries also a relatively high rate of treatment-related morbidity and mortality and is not suitable for unfit and/or older patients. Thus, there is an urgent need for less toxic and more effective therapies for AML patients.

Recently, the US Food and Drug Administration (FDA) has granted approval for several molecularly targeted therapies for AML [2–4]. Among them, venetoclax (VEN) is a selective BCL-2 inhibitor that has been shown to be highly effective in combination with low dose cytarabine (LDAC) or hypomethylating agents (HMA; i.e., azacytidine or decitabine) in inducing disease remission, with an overall response rate of approximately 60–70% in older and unfit AML patients [5–7]. However, despite these encouraging results, the majority of AML patients treated with VEN-based regimens eventually relapse. These treatment failures are often attributed to the persistence of leukemia stem cells (LSCs), a subpopulation of primitive AML cells with self-renewal and leukemia-initiating capacities, which have been shown to be highly refractory to conventional antileukemic treatments [8].

Recent reports have shown that metabolic mechanisms that support cell homeostasis of AML LSCs is profoundly different from those active in normal hematopoietic stem cells (HSCs) [9–11]. While HSCs utilize both oxidative phosphorylation (OXPHOS) and glycolysis, LSCs are deficient in glycolysis and highly depend on amino acid-driven OXPHOS for their basal energy requirements [12]. Of note, inhibition of BCL-2 has been shown to block OXPHOS and activate apoptosis in LSCs, thereby supporting the mechanistic activity of VEN on LSCs [10]. Interestingly, differential responses to VEN have been reported for LSCs from de novo AML patients and from relapsed/refractory (R/R) AML patients [11]. Whereas LSCs from de novo AML patients are sensitive to BCL-2 inhibition by VEN, LSCs from R/R AML patients are less sensitive to VEN, as they can also utilize fatty acid oxidation (FAO) as a rescuing pathway to fuel OXPHOS.

Nucleoside analogs have been long used as a backbone treatment of AML. Most of the compounds in this class of drugs exert their antileukemic activity by undergoing phosphorylation and incorporation into the DNA of malignant cells. Recently, we have developed a novel nucleoside analog, 8-chloro-adenosine (8-Cl-Ado) that is instead RNA-directed [13–15]. In cells, 8-Cl-Ado is phosphorylated to 8-chloro-adenosine mono- (8-Cl-AMP), di- (8-Cl-ADP) and triphosphate (8-Cl-ATP) metabolites. 8-Cl-Ado treatment resulted in a dramatic decline in intracellular ATP pool [13, 16, 17]. Under aerobic conditions, F_o_F_1_-ATP synthase (complex V of OXPHOS pathway) is the major source of bioenergetics in human cells. Mechanistic studies demonstrated that 8-Cl-ADP was a substrate and 8-Cl-ATP was an inhibitor of F_o_F_1_-ATP synthase [18]. These actions result in inhibition of OXPHOS pathway, cumulating in the diminishment of the ATP pool and activation of AMPK [19, 20]. This chlorinated adenosine has been shown to confer antineoplastic activities in hematological malignancies [13, 16, 17, 21] as well as in solid tumors [19, 20]. Specifically for AML, our preliminary data showed that 8-Cl-Ado can target LSCs while sparing HSCs [22], inhibits FLT3-ITD signaling [22] and has anti-neoplastic activity in vitro and in vivo [14, 15, 22–24]. The unique mechanism of action of 8-Cl-Ado as well as the preliminary encouraging results observed in an ongoing clinical trial of 8-Cl-Ado monotherapy in patients with refractory/relapsed (R/R) AML has led us to hypothesize that the combination of VEN and 8-Cl-Ado may provide synergistic antileukemic activity in AML cells. To this end, we show that 8-Cl-Ado can target FAO and synergizes with VEN to significantly decrease the oxygen consumption rate (OCR) and in turn OXPHOS in CD34-enriched AML cells. The net result is an enhanced antileukemic activity of the combination compared to each individual drug as demonstrated in vitro and in vivo.

## Methods

### Isolation of mononuclear cells from patient samples

Each patient specimen (see Additional file 1: Table S1 for primary AML blast cell sample information; see Additional file 1: Table S2 for patient treatment history) was transferred to a 50-mL conical tube and the volume was brought up to 25 mL using warm Dulbecco’s phosphate-buffered saline (DPBS; 1x) with 2% fetal bovine serum (FBS). The specimen was layered on top of 20 mL Ficoll-Paque Plus in a 50-mL conical tube. The tube was centrifuged at 300*g* for 32 min without breaking. The layer containing peripheral blood mononuclear cells and plasma was carefully transferred to a 50-mL conical tube and the volume was brought up to 50 mL with warm DPBS (1x). The tube was then centrifuged at 2.4 rpm for 8 min. The supernatant was discarded, and the pellet was resuspended in 10 mL of warm DPBS (1x). Cell number and viability were determined, and the sample was frozen. CD34^+^CD38− cells were then isolated using a magnetic bead selection protocol (Miltenyi Biotech, Germany).

### Cell lines and chemicals

MV4-11 (RRID:CVCL_0064) and KG1A (RRID:CVCL_1824) cells were purchased from the American Type Culture Collection (ATCC) and maintained in DMEM (Dulbecco modified Eagle medium) supplemented with 10% FBS and 100 units of penicillin/streptomycin at 37 °C with 5% CO_2_. Human cell lines purchased from ATCC more than 6 months prior to submission of this manuscript and not frozen at an early passage were authenticated using ATCCs’ human short tandem repeat DNA profiling authentication service. Morphology of cell lines was monitored routinely, and cell lines were routinely subjected to mycoplasma detection using a mycoplasma detection kit (Roche, Germany). Venetoclax and azacitidine were purchased from Selleckchem (Houston, Texas). 8-Chloro-adenosine was purchased from Tocris (Minneapolis, MN).

### Protein level measurement via immunoblot

Cells were washed and harvested in ice-cold phosphate-buffered saline (PBS) and subsequently lysed in RIPA buffer containing 10 mM protease inhibitor cocktail (Thermo Scientific, Lafayette, CO). For immunoblot analysis, 50 µg of each cell lysate was separated on NuPAGE 4–12% gradient gels (Invitrogen, Carlsbad, CA), and immunocomplexes were blotted with anti-p21 (Clone# F-5, Cat# sc-6246, Santa Cruz), anti-PCNA (Clone# F-2, Cat# 25280, Cell Signaling), anti-actin (Clone# C4, Cat# sc-47778, Santa Cruz), anti-IDI1 (Cat# ab97448, Abcam), anti-CD36 (Cat# ab252923, Abcam), anti-ACAT2 (Cat# ab131215, Abcam) and anti-TP53INP2 (Cat# ab273012, Abcam) antibodies and visualized with enhanced chemiluminescence reagent (Thermo Scientific, Lafayette, CO).

### Assessment of apoptosis using flow cytometry

The Annexin-V and DAPI double staining method was used. Cells were harvested and washed twice with Annexin-V binding buffer (BD Bioscience, San Jose, CA) and resuspended in 100 μL of the same buffer containing Annexin-V APC (BD Bioscience, San Jose, CA). Cells were then incubated in the dark at room temperature for 15 min, washed again and resuspended in 300 μL of buffer. DAPI (Sigma-Aldrich) was added immediately before analysis by an LSR II flow cytometer (BD Bioscience, San Jose, CA).

### DNA fragmentation analysis

Treated cells were lysed on ice for 60 min in 500 μL lysis buffer containing 0.02% SDS, 1% Nonidet P-40, and 0.2 mg/mL proteinase K in PBS. Genomic DNA was extracted using the phenol/chloroform method. The pellet was dissolved in 50 μL of TE buffer (supplemented with 10 mg/mL RNase) for 2 h at 37 °C. A total of 10 μg of DNA was loaded on a 2% agarose gel and visualized under UV light.

### FAO assay

Cultured cells were washed with HBSS and incubated with 200 µL of [^3^H]-palmitic acid (1 mCi/mL, PerkinElmer) bound to fatty-acid free albumin (100 µM; the ratio of palmitate:albumin is 2:1) and 1 mM l-cartinine. The complex was incubated for 2 h at 37 °C. The supernatant was collected after incubation and added to a tube containing 200 µL of cold 10% trichloroacetic acid. The tubes were centrifuged 10 min at 3000*g* at 4 °C, and aliquots of supernatants (350 µL) were removed, neutralized with 55 µL of 6 N NaOH and applied to an ion exchange column loaded with Dowex 1X2 chloride form resin (Sigma-Aldrich). The radioactive product was eluted with water. Flow-through was collected and radiation was quantified using liquid scintillation counting.

### Fatty acid uptake assay

Fatty acid uptake activities were analyzed following the manufacturer’s instructions (Abcam). Briefly, 10^5^ MV4-11, KG-1a or primary AML blast cells (*n* = 5) were seeded per well in a 96-well plate and treated for 24 h with the VEN (10 nM) plus 8-Cl-Ado (500 nM) combination, or with control. Fatty acid uptake was evaluated via the Free Fatty Acid Uptake Assay Kit (Fluorometric) (Cat# ab176768; Abcam). Briefly, after incubation with the drugs, cells were washed with phosphate-buffered saline (PBS), followed by a 1-h incubation in serum-free medium, and then treated with a fluorescent fatty acid mixture for an additional 30 min. The results were evaluated using a microplate fluorescence reader at 485/528 nm. The fluorescence signal from the control group was set to onefold for relative quantification.

### Seahorse assay

A total of 40,000 cells in 200 µL cell culture medium were seeded in each well of a XF-96-well cell culture microplate and cultured overnight at 37 °C in 5% CO_2_. As a negative control, three wells were kept devoid of cells and given only Seahorse media, which comprises basal XF media, 5.5 mM glucose, 1 mM sodium pyruvate and 4 mM glutamine (additionally, the pH was adjusted to 7.4). Twelve hours prior to running a plate, the Seahorse sensor cartridge was incubated with Seahorse Calibrant solution according to manufacturer’s protocol, in a 37 °C, CO_2_-free incubator. On the day of an assay, cells were washed and incubated with Seahorse media. The sensor cartridge was fitted onto the cell culture plate, which was then placed into a 37 °C, CO_2_-free incubator for 1 h. During the assay, which was run on the Seahorse XF96 Analyzer, the following inhibitors were injected sequentially, as is standard for the Cell Energy Test: oligomycin (1 mM), FCCP (0.5 mM).

### Measurement of intracellular 8-Cl-ATP

Intracellular concentrations of 8-Cl-ATP were measured using HPLC-tandem mass spectrometry as described previously [22].

### Animal experiments

NOD/SCID/γ chain^null^ mice (NSG, The Jackson Laboratory) were housed in micro-insulator cages in a pathogen-free condition and handled in laminar flow hoods. Six- to 8-week-old male NSG mice were i.v.-injected with 5 million luciferase-expressing MV4-11-Luc cells. Five days after tumor-cell injection, bioluminescence images of the mice were generated and the mice were separated in groups of 10 with equal average bioluminescence intensities, or randomly, if no luminescence signals were detected. Treatment commenced on day 7 after injection of the cancer cells. 8-Cl-Ado (50 mg/kg/day) or vehicle control (50% PEG300, 50% DMSO) for 8-Cl-Ado was administered by subcutaneously implanting an osmotic infusion pump (Model 2002, Alzet, Cupertino, CA) releasing 8-Cl-Ado at 50 mg/kg/day or vehicle control for 16 days. VEN (20 mg/kg) or vehicle control for VEN (10% ethanol, 60% Phosal 50 PG [Lipoid GmbH, Germany], 30% polyethylene glycol 400) was administered once daily via oral gavage, for 5 days per week. On days 5 and 21, the mice were injected intraperitoneally with luciferin and anesthetized with isoflurane, and the tumor burden (measured as photons) detected using a bioluminescence imaging system. Daily oral treatment with VEN or control solution was initiated at the same day the osmotic pumps were implanted. After 16 days, the 8-Cl-Ado- or control-vehicle-releasing osmotic pumps were replaced with new pumps. Survival was used as the endpoint measurement.

### Cell proliferation assay

Patient-derived, LSC-enriched blast cells or AML cell lines (20,000 cells/well in 100 μL) were transferred to 96-well plates previously prepared with 100 µL of 2 × concentrations of 8-Cl-Ado and/or VEN, as indicated in the main text and figures/figure legends. The cells were then incubated for 48 h prior to measurement of cell growth using the CellTiter Glo luminescent assay for primary cells or the CellTiter 96 AQueous One assay for cell lines (both assays, Promega).

### Combined drug effect analysis

For two-drug combination experiments, MV4-11, KG-1a and primary blast AML cells were treated with VEN, 8-Cl-Ado, or control vehicle for 48 h, as single agents as well as in combination, at constant ratios, on the basis of the previously calculated IC_50_ values for each drug. Quantitative analysis of dose–effect relationships was determined after measurement of cell growth using MTS or luminescence proliferation assays (Promega). Potential synergistic or additive effects were calculated using the software CompuSyn (Cambridge, UK). Isobolograms (not shown) and combination-index plots were created, and combination index (CI) values calculated. Drug synergism, addition, and antagonism effects are defined by CI values of < 0.9, 0.9–1.1, and > 1.1, respectively.

### Statistical analysis

To compare the means of 2 groups, results were generally compared by using an unpaired, two-tailed Student’s *t* test, with values from at least 2 independent experiments with triplicate determination, unless otherwise stated. Data are presented as mean ± standard error (S.E.), as indicated. The log-rank Kaplan–Meier survival test was used to compare the survival distributions of the different treatment groups, from time of cancer cell injection to death of the animals. *N* = 10 per group for the animal experiments. *p* < 0.05 and FDR < 0.05 was considered statistically significant; ns = not significant. For RNA-seq experiments, duplicate determination (2 replicates each) was used for MV4-11 and KG-1a cell lines, for all time points and treatments; patient samples were sequenced once (no replicates) for each treatment. All statistical analyses were conducted using SigmaPlot 12.5 (Systat Software, Chicago, Illinois). All statistical tests were two-sided.

### Gene set enrichment analysis (GSEA)

RNA sequencing (RNA-seq), RNA expression analysis and gene set enrichment analysis (GSEA) were performed as described previously [25] where the fatty acid metabolism and oxidative phosphorylation metabolism gene sets, containing 120 and 180 genes, respectively, were from the Molecular Signatures Database (MSigDB) Hallmark gene sets. The top 10 globally enriched pathways after GSEA analysis in each comparison were presented in a bubble plot, where color represents the significance, and the size the number of involved differentially expressed (DE) genes. Furthermore, to show the alteration of gene expression in the fatty acid metabolism pathway due to different treatments using a heatmap, the fold changes of core enrichment genes in log2 were collected from different time points from MV4-11 and KG-1a cell lines and from different primary AML blasts (i.e., patient #1 and patient #2).

## Results

### 8-chloro-adenosine attenuates fatty acid oxidation and oxidative phosphorylation in AML

**Fig. 1.**
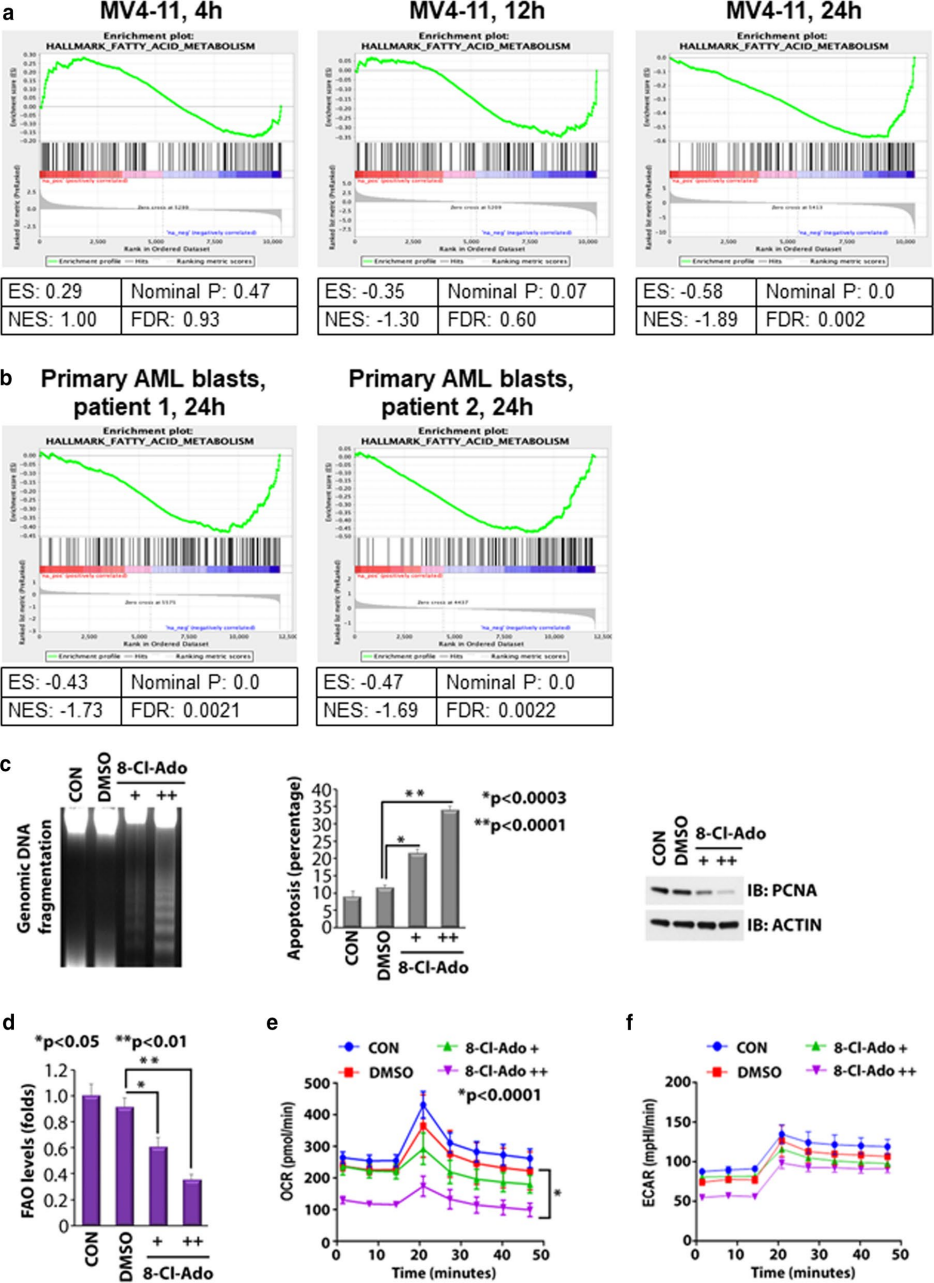
8-Cl-Ado attenuates FAO and OXPHOS in AML. **a** + **b** Gene set enrichment analysis (GSEA) graphs of genes involved in fatty acid metabolism (Hallmark fatty acid metabolism) upon treatment of MV4-11 AML cells for 4, 12, and 24 h with 250 nM 8-Cl-Ado (**a**) and primary AML blasts for 24 h with 500 nM 8-Cl-Ado (**b**) versus control. ES, enrichment score; NES, normalized enrichment score; FDR, false discovery rate. *N* = 2 for MV4-11; *N* = 1 for primary AML blasts. **c** Effects of 8-Cl-Ado on apoptosis of CD34+CD38− AML blasts. Cells were treated with 500 nM (+) and 1 µM (++) 8-Cl-Ado for 48 h. Left, fragmentation of genomic DNA, as measured by gel electrophoresis. Middle, apoptosis as measured by Annexin V/DAPI staining and flow cytometry. Right, measurement of PCNA by western blotting. **d** Effects of 8-Cl-Ado on FAO levels of CD34 +CD38− AML blasts. The cells were treated for 24 h as described in **c** and FAO assay results are presented as fold change, compared to control. **e**, **f** Effects of 8-Cl-Ado on levels of OCR and ECAR of CD34 +CD38− AML blasts. The cells were treated for 24 h as described in **c**, and levels of OCR (**e**) and ECAR (**f**) were measured using the Seahorse XF cell energy phenotype test kit

We previously reported preclinical activity of 8-Cl-Ado against FLT3-ITD AML blast cells and LSCs [22]. In contrast to other clinically used nucleoside analogs that contain deoxyribose or arabinose moieties, 8-Cl-Ado contains ribose sugar and therefore is predominantly incorporated into newly transcribed RNA, thereby causing chain termination and resulting in a decline in cellular proteins. Herein, we tested the activity of the drug on proteins that mediate FAO, a potential mechanism of resistance to VEN. To this end, we first treated MV4-11 AML cells for 4–24 h with 250 nM 8-Cl-Ado, a previously established IC_50_ (IC_50_ at *t* = 72 h) for this cell line, and then subjected these cells to mRNA sequencing. Gene set enrichment analysis (GSEA) showed that 8-Cl-Ado caused downregulation of a gene set involved in fatty acid metabolism in the treated cells at 12 and 24 h after start of treatment (Fig. 1a), as compared to vehicle-treated controls. After 24 h, suppression of fatty acid metabolism was highly significant (false discovery rate, FDR, 0.002) (Fig. 1a, right). Treatment of primary blast cells from two patients with AML with 500 nM 8-Cl-Ado for 24 h also resulted in highly significant negative enrichment of the fatty acid metabolism gene set, as assessed by GSEA (Fig. 1b). Downregulation of fatty acid metabolism by GSEA was also observed in the KG-1a cell line treated for 12 and 24 h with 500 nM 8-Cl-Ado, although only the 12 h time point was significant (Additional file 1: Figure S1).

Given that 8-Cl-Ado downregulates fatty acid metabolism in AML cell lines and primary blasts as indicated by GSEA, we then validated the effects of 8-Cl-Ado on metabolic and cellular activity in CD34+/CD38− primary AML blasts. As shown in Fig. 1c, DNA fragmentation, apoptosis and inhibition of cell proliferation were all detectable at 500 nM 8-Cl-Ado and further increased at 1 μM, after 48 h of treatment. We then performed real-time metabolic assays in CD34+/CD38− AML blasts treated with 500 nM and 1 μM 8-Cl-Ado, for 24 h. The oxidation rate of ^3^H-palmitic acid was used to measure fatty acid oxidation (FAO) levels (Fig. 1d). The Agilent Seahorse XF96 analyzer was used to measure the oxygen consumption rate (OCR), indicative of OXPHOS (Fig. 1e) and the extracellular acidification rate (ECAR), which designates glycolysis (Fig. 1f). Treatment of CD34-enriched AML blasts with 500 nM or 1 μM 8-Cl-Ado resulted in significantly decreased FAO (Fig. 1d). OXPHOS was significantly blunted in AML blasts treated with 1 μM 8-Cl-Ado (*p* < 0.0001) but not with a concentration of 500 nM (Fig. 1e). No significant effects were seen on ECAR with either 8-Cl-Ado concentrations (Fig. 1f).

### Venetoclax and 8-chloro-adenosine synergize in the inhibition of oxidative phosphorylation in AML

**Fig. 2.**
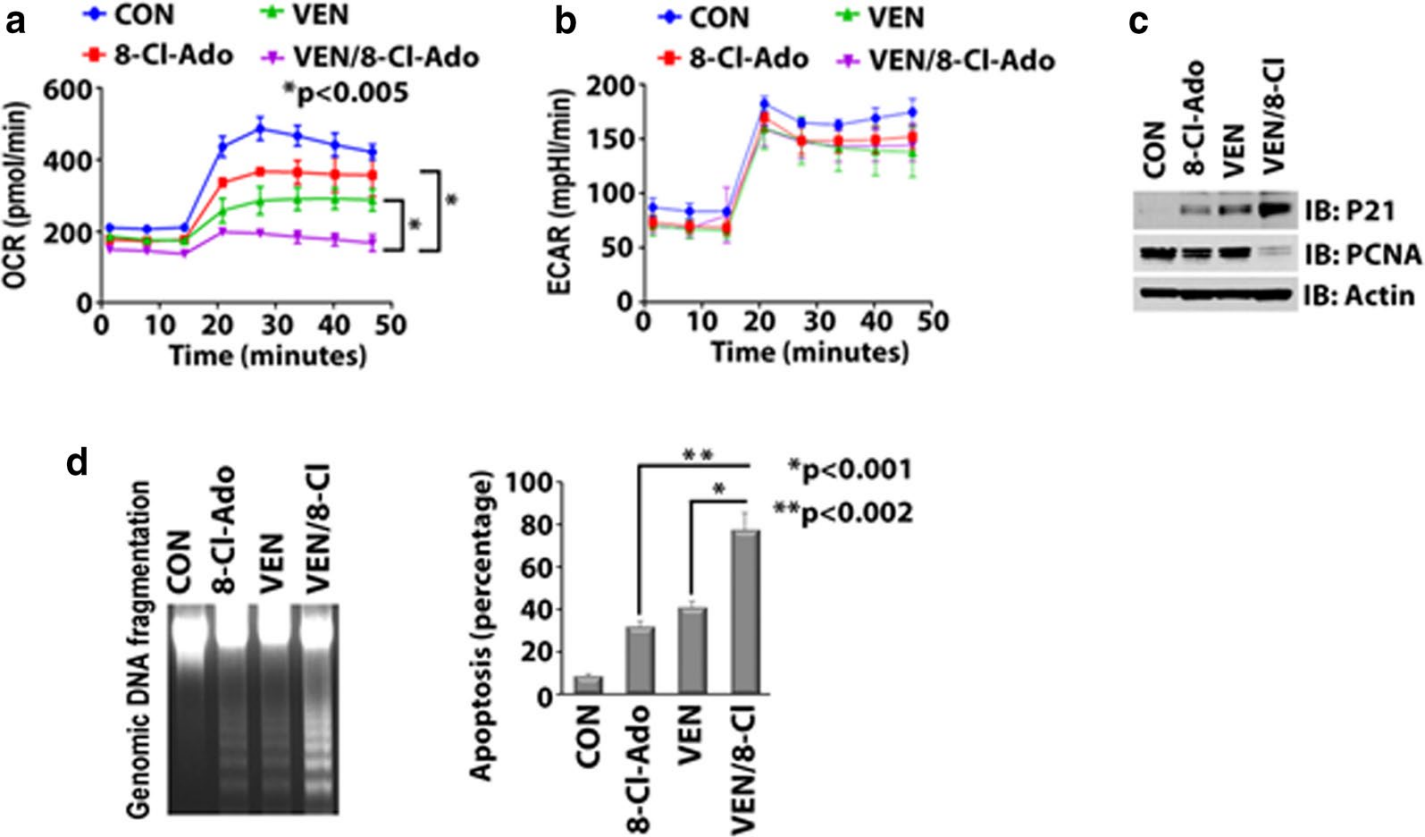
8-Cl-Ado and VEN synergize in inhibition of OXPHOS metabolism in AML. **a** + **b** Effects of combined treatment of 8-Cl-Ado and VEN on levels of OCR and ECAR in CD34+CD38− AML blasts. Cells were treated with control, 500 nM 8-Cl-Ado, 10 nM VEN or 8-Cl-Ado plus VEN for 24 h. Levels of OCR (**a**) and ECAR (**b**) were measured using the Seahorse XF cell energy phenotype test kit. **c** + **d** CD34+CD38− AML blast cells were treated for 48 h as described in **a** + **b**. **c** Effects of combined treatment of 8-Cl-Ado and VEN on levels of p21 and PCNA protein expression. Each lysate was immunoblotted with indicated antibodies. **d** Effects of combined treatment of 8-Cl-Ado and VEN on apoptosis levels. Left, genomic DNA was isolated and DNA fragmentation is shown. Right, apoptosis levels as measured by Annexin V/DAPI staining using flow cytometry

Having demonstrated an 8-Cl-Ado-mediated inhibition of FAO and OXPHOS, we next examined whether addition of 8-Cl-Ado to VEN would augment the antileukemic activity in LSC-enriched AML blasts. Firstly, we showed that treatment of CD34+CD38− cells with 10 nM VEN and 500 nM 8-Cl-Ado for 24 h completely inhibited OCR, in contrast to effects from single agents (*p* < 0.005) (Fig. 2a), while ECAR was not significantly affected (Fig. 2b). Of note, the VEN/8-Cl-Ado combination increased activation of the cyclin-dependent kinase inhibitor p21, decreased the proliferation marker PCNA (Fig. 2c), augmented DNA fragmentation and enhanced apoptosis in the CD34+/CD38− AML blasts after 48 h of treatment (Fig. 2d).

**Fig. 3:**
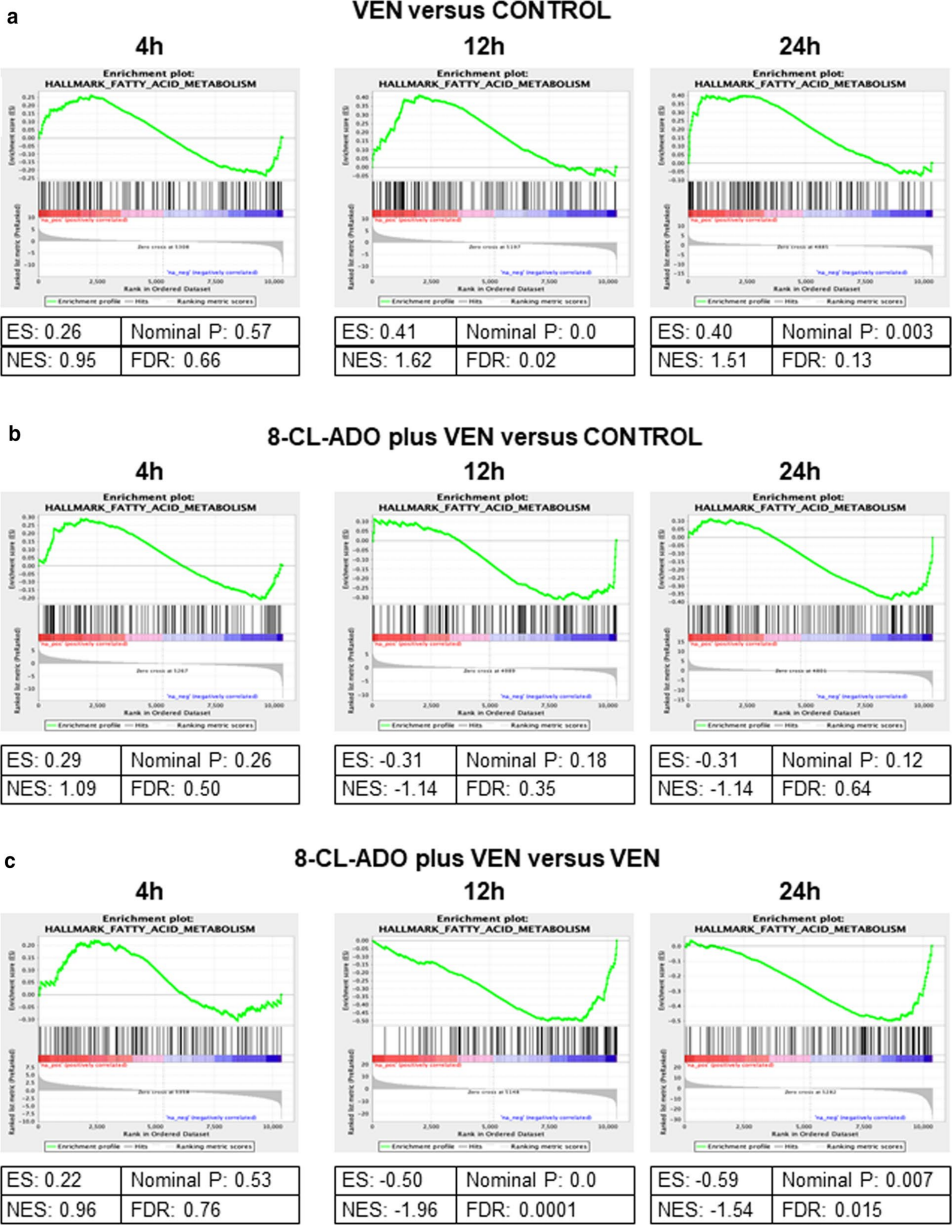
GSEA shows that 8-Cl-Ado and VEN synergize in inhibition of fatty acid metabolism in AML. Gene set enrichment analysis (GSEA) graphs of genes involved in fatty acid metabolism (Hallmark fatty acid metabolism) upon treatment of MV4-11 AML cells with 10 nM VEN or control (**a**), 250 nM 8-Cl-Ado plus 10 nM VEN or control (**b**), for 4 to 24 h. **c** Fatty acid metabolism GSEA for direct comparison of VEN/8-Cl-Ado versus VEN alone. Cells were treated as described above. *N* = 2. *ES* enrichment score, *NES* normalized enrichment score, *FDR* false discovery rate

Interestingly, when we performed GSEA on MV4-11 AML cells treated with VEN/8-Cl-Ado, we found that VEN treatment alone significantly increased expression of the fatty acid metabolism gene set at 12 h (Fig. 3a), whereas the VEN/8-Cl-Ado combination decreased the expression of this gene set, as indicated by the negative enrichment scores ES and NES (Fig. 3b). Direct comparison of the VEN/8-Cl-Ado combination and VEN alone indicated the statistically significant decreased activity of the fatty acid metabolism pathway at 12 h and 24 h in the VEN/8-Cl-Ado combination, as indicated by the negative enrichment scores ES and NES, nominal *p* value and FDR (Fig. 3c). To show the alteration of gene expression in the fatty acid metabolism pathway due to different treatments of MV4-11, KG-1a and primary AML blasts (patient #1 and patient #2) using heatmaps, we display the fold changes of core enrichment genes in log2 in Additional file 1: Figure S2. Core enrichment genes for MV4-11 are shown in Additional file 1: Figure S2A; those for KG-1a and primary AML blasts are presented in Additional file 1: Figure S2B. It is interesting that the combination treatment in MV4-11 cells even had strong activation of the fatty acid metabolism pathway at 4 h. A similar effect was observed in the OXPHOS pathway activity, although the level of statistical significance is somewhat lower (Additional file 1: Figure S3).

Direct comparison of VEN/8-Cl-Ado treatment versus VEN treatment alone by GSEA revealed negative enrichment of the gene sets for fatty acid metabolism (Additional file 1: Figure S4) and OXPHOS (Additional file 1: Figure S5) also in KG-1a AML cells (12 h and 24 h) and in primary AML blasts (24 h), with higher significance in the KG-1a cell line.

**Fig. 4:**
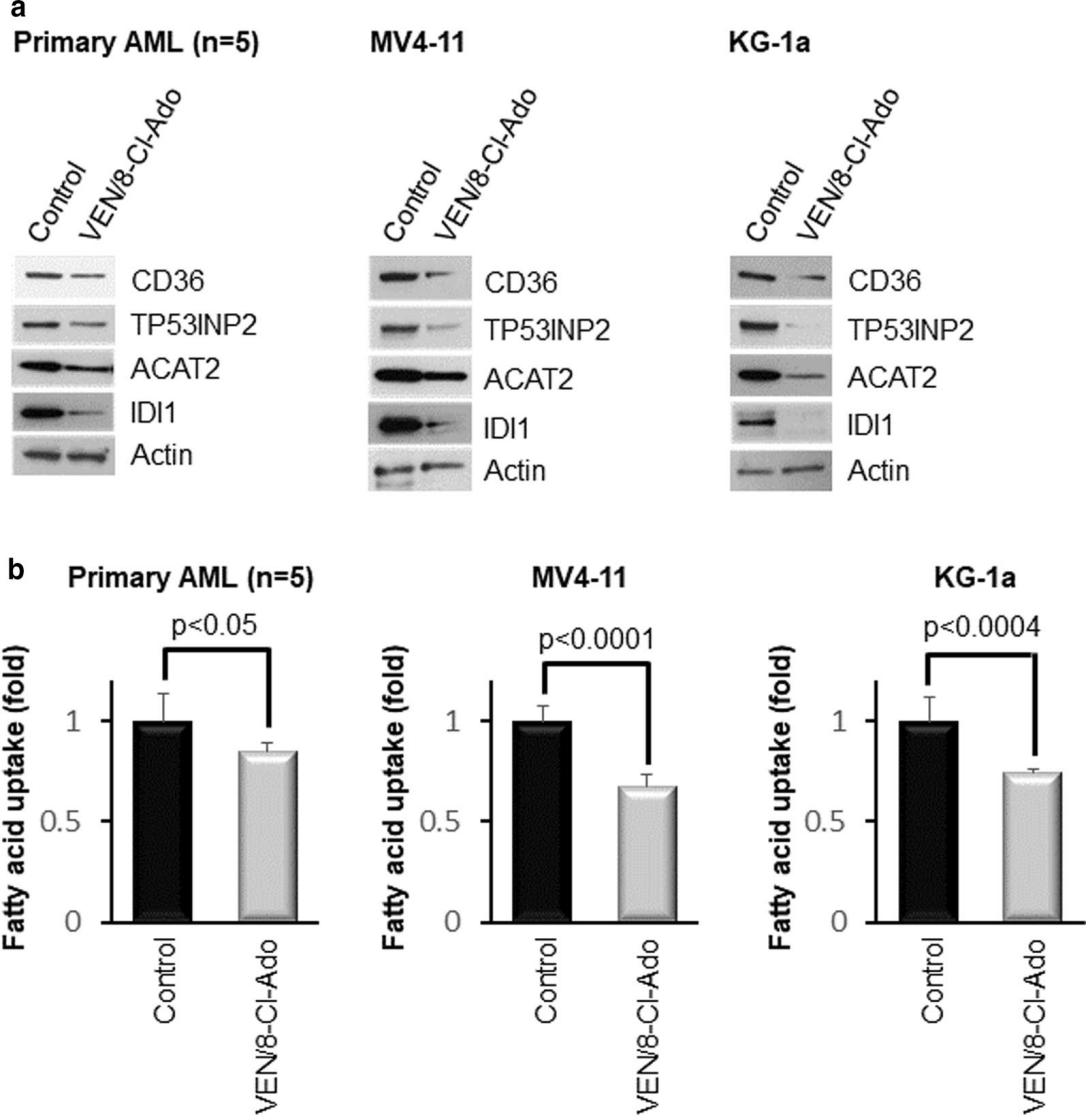
The VEN/8-Cl-Ado combination downregulates proteins involved in fatty acid metabolism and inhibits fatty acid uptake in AML cells. **a** Western blotting with antibodies against proteins with decreased mRNA expression, as assessed by GSEA for fatty acid metabolism of the MV4-11 cell line treated with VEN/8-Cl-Ado for 24 h. Primary AML blasts (pool of cells from 5 patients) and MV4-11 and KG-1a cells were treated for 24 h with 500 nM 8-Cl-Ado and 10 nM VEN, or control, prior to cell lysis and Western blotting with antibodies against CD36, TP53INP2, ACAT2, IDI1 and actin (loading control). **b** Fatty acid uptake assay was performed in primary AML blasts and KG-1a and MV4-11 cells treated for 24 h as described in **a** followed by fatty acid uptake assay

To further verify the effect of the VEN/8-Cl-Ado combination treatment on AML cells, we performed Western blotting with antibodies against proteins with decreased mRNA expression, as assessed by GSEA, for fatty acid metabolism of the MV4-11 cell line treated with 250 nM 8-Cl-Ado for 24 h. For this intent, we treated primary AML blasts (pool of cells from 5 patients) and MV4-11 and KG-1a cells for 24 h with 500 nM 8-Cl-Ado and 10 nM VEN, or control, prior to cell lysis and Western blotting analysis. As shown in Fig. 4a, the expression levels of 4 selected proteins involved in fatty acid metabolism, CD36 (fatty acid translocase, imports fatty acids into cells) [26], TP53INP2 (tumor protein p53-inducible nuclear protein 2, involved in transcription of adipogenic genes and in autophagy) [27], ACAT2 (acetyl-CoA acetyltransferase 2, involved in cholesterol absorption, converts cholesterol and fatty acid to cholesteryl esters) [28] and IDI1 (isopentenyl pyrophosphate isomerase 1, a core enzyme in isoprenoid biosynthesis) [29] were decreased in all samples investigated. Because CD36 is involved in regulating fatty acid uptake [26], we performed a fatty acid uptake assay to investigate the biological effect of CD36 downregulation upon treatment of AML cells with the VEN/8-Cl-Ado combination. As shown in Fig. 4b, 24 h treatment with VEN (10 nM) plus 8-Cl-Ado (500 nM) significantly downregulated fatty acid uptake in primary AML blasts (pool of blast cells from 5 patients) and MV4-11 and KG-1a cells, as compared to control-treated cells.

Since the VEN/8-Cl-Ado combination might also target other pathways, in addition to fatty acid metabolism, we analyzed the full set of DE genes of MV4-11 cells treated with VEN/8-Cl-Ado for 12 h and 24 h, and the top 10 globally enriched pathways after GSEA analysis in each comparison are presented in a bubble plot graph, where color represents the significance, and the size the number of involved DE genes (Additional file 1: Figure S6). The bubble plot graphs show that metabolic pathways involved in cell energy production (e.g., fatty acid metabolism, glycolysis, oxidative phosphorylation) were negatively enriched at 12 h (Additional file 1: Figure S6A) and/or 24 h (Additional file 1: Figure S6B). Further, critical pathways involved in cell cycle regulation (e.g., G2M-checkpoint, Myc-targets and E2F-targets) were negatively enriched at *t* = 12 h and *t* = 24 h. In parallel, we detected upregulation of the pro-apoptotic p53 pathway at *t* = 24 h.

### 8-chloro-adenosine enhances the antileukemic activity of venetoclax in vitro and in vivo

**Fig. 5:**
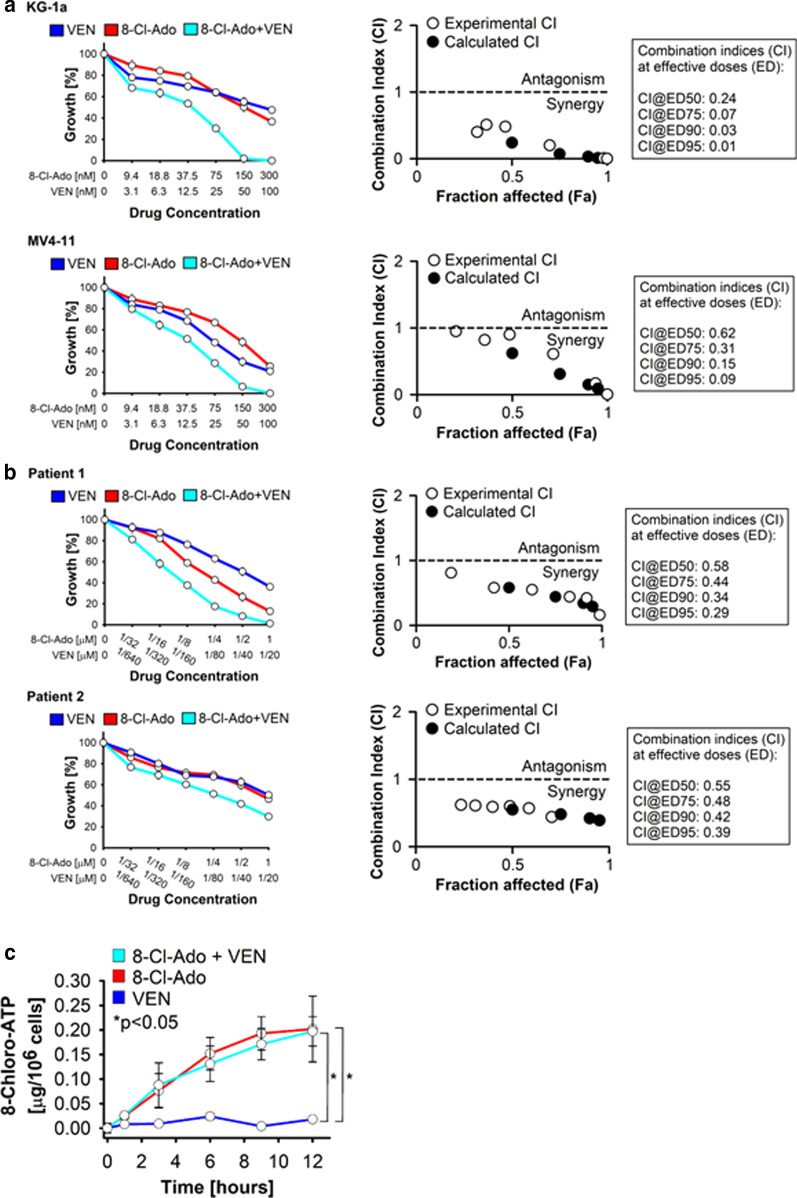
Effect of VEN plus 8‐Cl‐Ado combinatorial treatment on growth of AML cells in vitro. Effect of VEN plus 8‐Cl‐Ado combinatorial treatment on the growth of AML cell lines KG-1a and MV4-11 (**a**) and primary CD34+CD38− AML blasts (**b**). Cells were treated for 48 h with control vehicle, individual drugs or with the 2-drug combination at constant drug ratios, on the basis of previously established IC_50_ values, followed by measurement of cell proliferation. Drug synergy was analyzed using the CalcuSyn program. Data are presented as mean ± SE, with quadruplicate determination. Results from one representative experiment of 2 independent experiments are shown. Combination index blots and combination indices (CI) are presented. Drug synergism, addition, and antagonism effects are defined by combination index values of < 1.0, 1.0, and > 1.0, respectively. **c** Intracellular concentration of 8‐Cl‐ATP in MV4-11 cells (2 × 10^5^ cells/mL) treated with 250 nM 8‐Cl‐Ado, 10 nM VEN, or both, for up to 12 h. Results from one representative experiment (in duplicates) of 2 independent experiments are shown

The VEN/8-Cl-Ado combination synergistically inhibited growth of the AML cell lines KG-1a and MV4-11 (Fig. 5a) and primary patient blast cells (Fig. 5b), after 48 h. The experimental and calculated combination indices (CIs) (right panels in Fig. 5a, B) demonstrate VEN/8-Cl-Ado drug synergy in the cell lines and in the primary AML blasts. The CIs were < 1 for the drug combination using concentrations predicted to inhibit 50, 75, 90 or 95 percent of cell growth (ED50-ED95). As the anticancer activity of 8-Cl-Ado depends on an efficient intracellular conversion of the parent drug into 8-Cl-AMP and subsequent phosphorylation into the cytotoxic metabolite, 8-Cl-ATP, we then measured the 8-Cl-ATP intracellular accumulation in MV4-11 AML cells treated up to 12 h with 8-Cl-Ado, VEN or both. Addition of VEN to 8-Cl-Ado did not significantly alter 8-Cl-ATP accumulation (Fig. 5c), suggesting that the two drugs may synergize by independent antileukemic mechanisms.

**Fig. 6.**
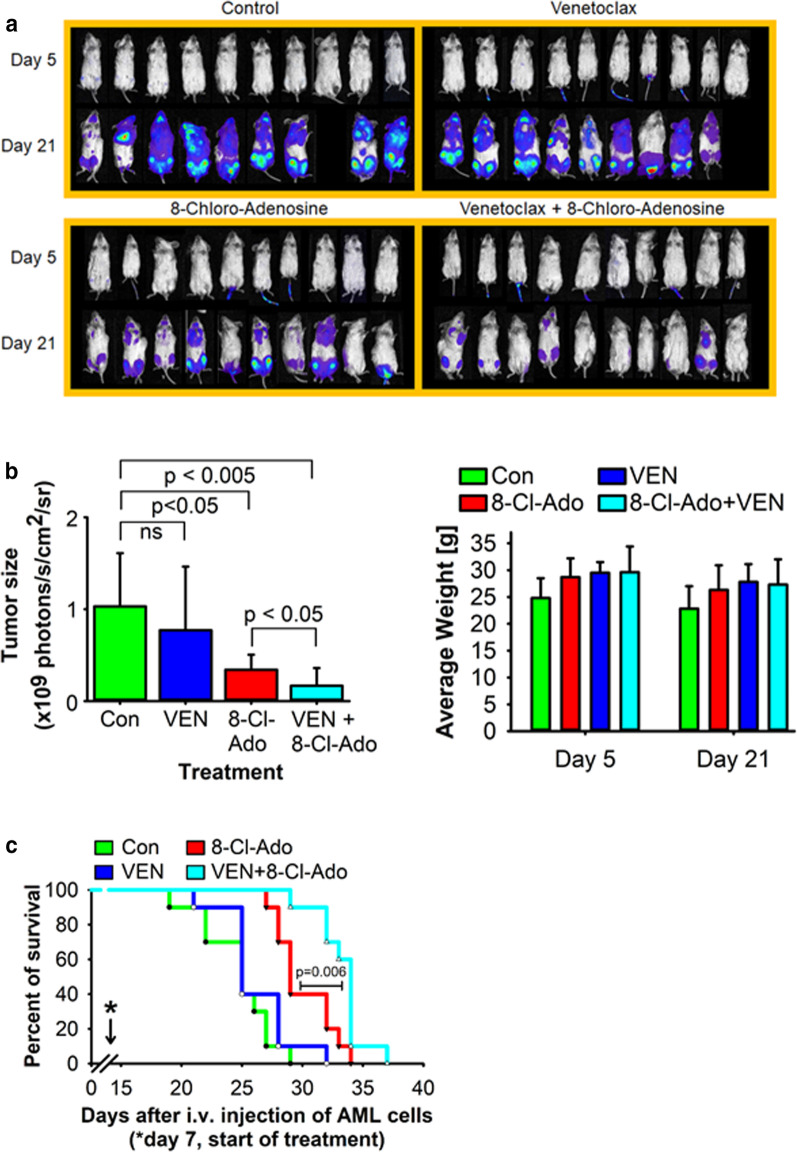
8‐Cl‐Ado enhances the antileukemic effect of VEN in vivo. MV4-11‐Luc AML cells (5 × 10^6^) were i.v.-injected in NSG mice 7 days before start of treatment with vehicle control, 50 mg/kg/day 8-Cl-Ado (daily), 20 mg/kg/day VEN (5 days per week) or 8-Cl-Ado plus VEN. **a** Bioluminescence images obtained on days 5 and 21. **b** Left, Tumor burden on day 21, as assessed by analysis of bioluminescence images. Right, Average weight of animals obtained on days 5 and 21. **c** Days of survival were quantified using the Kaplan–Meier log rank survival test

To test the VEN/8-Cl-Ado combination in vivo, we then engrafted NSG mice with FLT3‐ITD MV4-11-Luc AML cells 7 days prior to start of treatment with vehicle control, 50 mg/kg/day daily 8-Cl-Ado (via Alzet osmotic pump), 20 mg/kg/day 5x/week oral VEN, or 8-Cl-Ado plus VEN. Bioluminescence measured at days 5 and 21 post-engraftment of the leukemic cells (Fig. 6a) revealed a decreased leukemia burden in mice treated with 8-Cl-Ado (*p* < 0.05) and 8-Cl-Ado/VEN (*p* < 0.005) but not in those treated with VEN alone, at day 21 (Fig. 6b, left). The VEN/8-Cl-Ado combination treatment group significantly decreased the leukemia burden compared to 8-Cl-Ado alone (*p* < 0.05) (Fig. 6b, left). Compared to the control group, no significant weight changes were detected in these treatment groups, suggesting that the combination at this concentration did not have an enhanced risk of toxicity (Fig. 6b, right). While VEN as a single agent did not change survival compared with vehicle, both 8-Cl-Ado- and 8-Cl-Ado/VEN extended survival. However, 8-Cl-Ado/VEN induced a significantly longer survival compared with that of 8-Cl-Ado alone (Fig. 6c; *p* = 0.006). Of note, when the concentration of VEN was increased to 100 mg/kg/day (with 8-Cl-Ado at 50 mg/kg/day), we observed weight loss in the animals treated with the VEN/8-Cl-Ado combination, suggesting the potential for toxicity for the 2-drug combination at elevated VEN concentrations (not shown).

### 8-chloro-adenosine and venetoclax inhibit fatty acid oxidation in relapsed AML

**Fig. 7:**
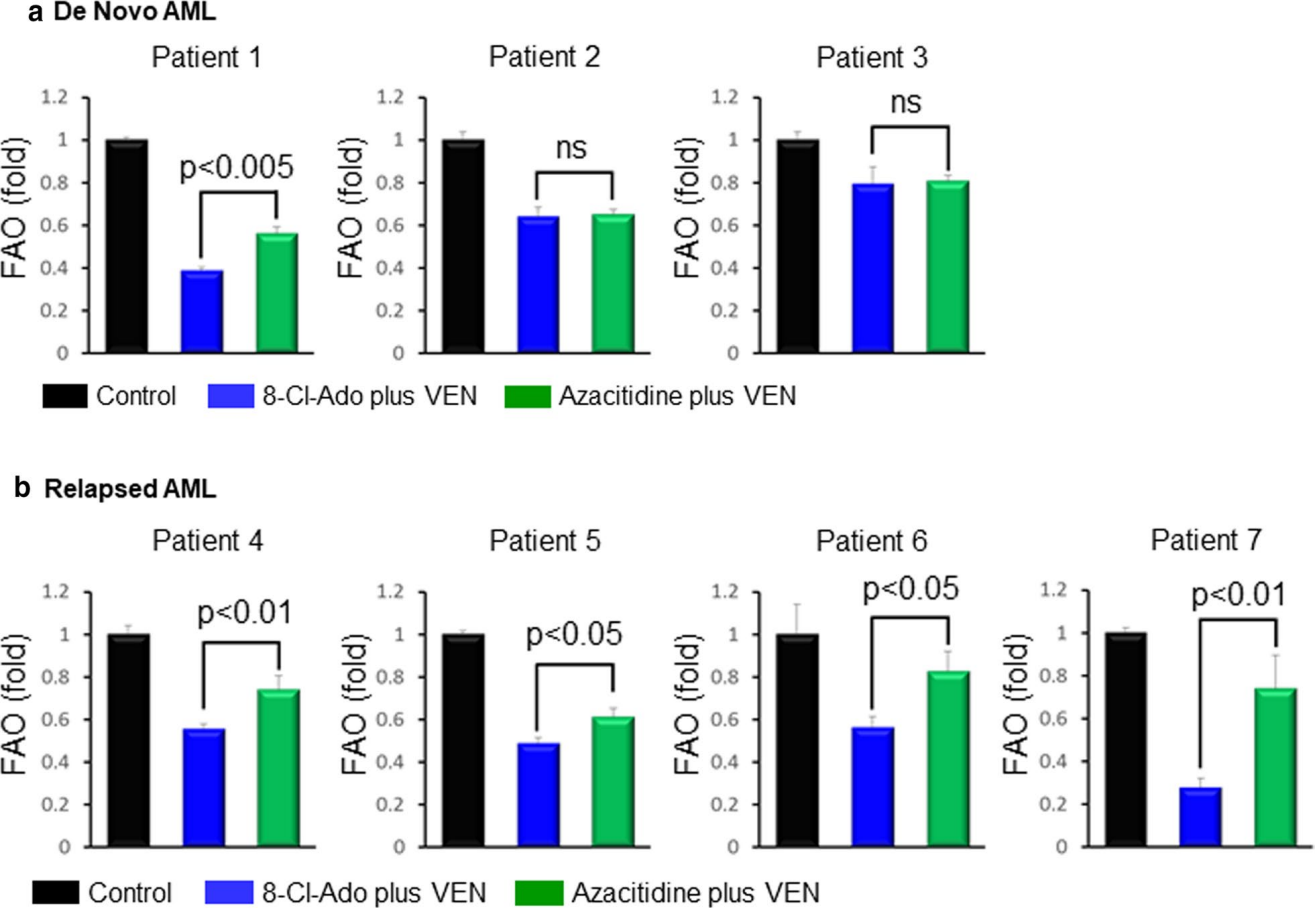
Fatty acid oxidation of de novo and relapsed primary AML cells treated with VEN/8-Cl-Ado and VEN/AZA versus control. Fatty acid oxidation of de novo (**a**) and relapsed (**b**) primary AML cells treated for 24 h with 10 nM VEN plus 500 nM 8-Cl-Ado or 10 nM VEN plus 5 µM AZA

Jones et al. have recently shown that VEN in combination with the hypomethylating agent azacitidine (AZA) reduces OXPHOS in an amino acid-dependent manner, in LSCs isolated from patients with de novo AML [11], thus providing a possible mechanism for the high efficacy seen with this combination in patients with de novo AML. However, studies in patients with relapsed/refractory AML have shown only minor efficacy for the VEN/AZA combination [30], and Jones et al. have demonstrated that LSCs from patients with relapsed/refractory AML can escape loss of amino acid/(BCL-2)-dependent OXPHOS by increasing fatty acid metabolism-driven OXPHOS [11]. Since we have shown that the VEN/8-Cl-Ado drug combination inhibits fatty acid metabolism and OXPHOS in AML cells, we asked how the VEN/8-Cl-Ado drug combination affects FAO in AML blasts compared to the VEN/AZA combination. For this purpose, we first treated MV4-11, KG-1a and primary AML blasts (pool of AML blasts from 5 patients) for 24 h with 10 nM VEN in combination with 500 nM 8-Cl-Ado and with 10 nM VEN in combination with 5 µM AZA. As shown in Additional file 1: Figure S7, both VEN/AZA and VEN/8-Cl-Ado inhibited FAO in MV4-11, KG-1a and in primary AML blast cells, as compared to untreated control cells. Importantly, the VEN/8-Cl-Ado combination caused significantly lower levels of FAO compared to the VEN/AZA combination. To compare the effects of VEN/AZA with VEN/8-Cl-Ado on FAO in relapsed versus de novo AML, we treated AML blast cells from 3 patients with de novo AML and 4 patients with relapsed/refractory AML with 10 nM VEN in the presence of 500 nM 8-Cl-Ado or 5 µM AZA, for 24 h. As shown in Fig. 7, both VEN combination treatments inhibited FAO in all 7 patient blast cells, to varying degrees. Statistically significant differences between VEN/AZA and VEN/8-Cl-Ado were seen in 5 of the 7 patient samples. Importantly, the VEN/8-Cl-Ado combination, as compared to the VEN/AZA combination, showed significantly greater inhibition of FAO in each of these 5 patient samples. Moreover, the VEN/8-Cl-Ado combination also showed significantly decreased levels of FAO in all blasts from the 4 relapsed AML patients, when compared to VEN/AZA (Fig. 7b). In contrast, only 1 in 3 primary blast samples from de novo AML patients investigated showed significantly decreased FAO levels by VEN/8-Cl-Ado (Fig. 7a) when compared to VEN/AZA, while no significant differences were seen between VEN/AZA and VEN/8-Cl-Ado for the remaining 2 samples. These results demonstrate that the VEN/8-Cl-Ado combination targets FAO in primary blasts from patients with relapsed/refractory AML.

## Discussion

Combinations of VEN with either an HMA drug or low dose cytarabine have been FDA-approved for newly diagnosed older, unfit patients with AML and are also increasingly considered for fit patients. We and others recently showed that VEN in combination with HMAs augments oxidative stress in AML cells [31]. Although VEN-based combinations frequently induce AML response, the majority of patients treated with these regimens eventually relapse, likely because of LSC resistance. LSC homeostasis has been shown to depend on amino acid-driven OXPHOS. Recently, it has been reported that, whereas the homeostasis of de novo AML LSCs depends on amino acid-driven OXPHOS, LSCs isolated from patients with relapsed AML appear to be less dependent on amino acid consumption and more on FAO to fulfill their energy production needs. Therefore, novel agents targeting FAO in LSCs may improve the activity of VEN-based regimens.

We have previously reported that the novel RNA-nucleoside 8-Cl-Ado not only targets LSCs [22] but rapidly diminishes the bulk of the circulating AML blast cells (unpublished results from phase I clinical trial with 8-Cl-Ado; NCT02509546). Herein, we demonstrate that VEN in combination with 8-Cl-Ado synergistically inhibits growth of AML cells, including primary CD34 + blasts. Importantly, we showed that 8-Cl-Ado significantly attenuated OXPHOS in AML blasts by inhibiting FAO. Of note, LSCs from R/R AML patients are known to utilize both FAO and amino acid-dependent OXPHOS but can switch to FAO for their metabolic requirements, thus bypassing dependence on amino acid-driven OXPHOS [11]. Utilization of FAO instead of amino acid-driven OXPHOS provides a possible explanation for increased resistance to VEN in blasts and LSCs from R/R AML patients [11]. We have shown in AML cell lines and in primary AML blast cells that the VEN/8-Cl-Ado treatment downregulates important proteins involved in fatty acid metabolism. More specifically, we consistently detected downregulation of acetyl-CoA acetyltransferase 2 (ACAT2) and fatty acid translocase (CD36), two proteins involved in cholesterol absorption and metabolism [28] and import and metabolism of fatty acids [26], respectively. Of note, CD36 is linked to fueling tumor metastasis and drug resistance by increasing FAO in cancer and is a potential target for cancer treatment [26]. Importantly, we show that the VEN/8-Cl-Ado combination is significantly more potent in inhibition of FAO in primary blasts from relapsed AML patients when compared to the VEN/AZA combination. In fact, our GSEA data suggest that some AML cells exposed to VEN might compensate for loss of the BCL-2/amino acid/OXPHOS pathway by upregulating fatty acid metabolism. The combination of 8-Cl-Ado with VEN therefore results in a completely inhibited OXPHOS in the CD34 + AML blasts. Because the VEN/8-Cl-Ado treatment likely also affects other pathways in addition to fatty acid metabolism, we have performed additional analyses in AML cells to detect changes in globally enriched pathways upon VEN/8-Cl-Ado treatment. The top 10 negative enriched pathways after GSEA analysis included pathways required for cell energy production, including glycolysis, OXPHOS and fatty acid metabolism, thus supporting our hypothesis that the VEN/8-Cl-Ado combination targets metabolic vulnerabilities in AML. Other pathways that were negatively enriched after 12 h and 24 h included those involved in cell cycle regulation (e.g., G2M-checkpoint, Myc-targets, and E2F-targets). In parallel, we detected upregulation of the pro-apoptotic p53 pathway at *t* = 24 h. Lastly, we have previously reported strong anti-leukemic activity of 8-Cl-Ado in an orthotopic mouse model of AML, using the rapidly growing FLT3-ITD-positive AML cell line MV4-11 [22]. The addition of VEN to 8-Cl-Ado in this aggressive AML mouse model resulted in significantly increased additional survival, supporting the potential clinical utility of this combination.

## Conclusion

In summary, our data suggest that enhanced antileukemic effects may be achieved by combining 8-Cl-Ado with VEN to ensure maximum inhibition of FAO and OXPHOS and to eradicate AML progenitor cells. A phase I/II clinical trial with VEN plus 8-Cl-Ado in patients with R/R AML will soon be initiated at our institution.

## Supplementary Information

**Additional file 1:** Supplemental figures and tables.

## Data Availability

The bioinformatics raw data/analyzed raw data used in the current study are available from the corresponding authors on reasonable request and at: Link to GEO DataBase.

## Abbreviations

8-Cl-Ado: 8-Chloro-adenosine
alloSCT: Allogeneic stem cell transplantation
AML: Acute myeloid leukemia
ATCC: American Type Culture Collection
AZA: Azacitidine
BM: Bone marrow
ECAR: Extracellular acidification rate
FAO: Fatty acid oxidation
FDA: Food and Drug Administration
GSEA: Gene set enrichment analysis
HMA: Hypomethylating agents
HSC: Hematopoietic stem cells
LDAC: Low dose cytarabine
LSC: Leukemia stem cells
NSG: NOD/SCID/γ chain^null^
OXPHOS: Oxidative phosphorylation
R/R: Relapsed/refractory
VEN: Venetoclax

## References

[1] Siegel RL, Miller KD, Fuchs HE, Jemal A (2021). Cancer statistics. CA Cancer J Clin.

[2] Krauss AC, Gao X, Li L, Manning ML, Patel P, Fu W, Janoria KG, Gieser G, Bateman DA, Przepiorka D (2019). FDA Approval Summary: (Daunorubicin and Cytarabine) liposome for injection for the treatment of adults with high-risk acute myeloid leukemia. Clin Cancer Res.

[3] Norsworthy KJ, Ko CW, Lee JE, Liu J, John CS, Przepiorka D, Farrell AT, Pazdur R (2018). FDA Approval Summary: mylotarg for treatment of patients with relapsed or refractory CD33-positive acute myeloid leukemia. Oncologist.

[4] Bohl SR, Bullinger L, Rucker FG (2019). New targeted agents in acute myeloid leukemia: new hope on the rise. Int J Mol Sci.

[5] DiNardo CD, Pratz K, Pullarkat V, Jonas BA, Arellano M, Becker PS, Frankfurt O, Konopleva M, Wei AH, Kantarjian HM (2019). Venetoclax combined with decitabine or azacitidine in treatment-naive, elderly patients with acute myeloid leukemia. Blood.

[6] Pollyea DA, Amaya M, Strati P, Konopleva MY (2019). Venetoclax for AML: changing the treatment paradigm. Blood Adv.

[7] DiNardo CD, Jonas BA, Pullarkat V, Thirman MJ, Garcia JS, Wei AH, Konopleva M, Dohner H, Letai A, Fenaux P (2020). Azacitidine and venetoclax in previously untreated acute myeloid leukemia. N Engl J Med.

[8] Felipe Rico J, Hassane DC, Guzman ML (2013). Acute myelogenous leukemia stem cells: from Bench to Bedside. Cancer Lett.

[9] Gilliland DG, Jordan CT, Felix CA (2004). The molecular basis of leukemia. Hematol Am Soc Hematol Educ Program.

[10] Lagadinou ED, Sach A, Callahan K, Rossi RM, Neering SJ, Minhajuddin M, Ashton JM, Pei S, Grose V, O'Dwyer KM (2013). BCL-2 inhibition targets oxidative phosphorylation and selectively eradicates quiescent human leukemia stem cells. Cell Stem Cell.

[11] Jones CL, Stevens BM, D'Alessandro A, Reisz JA, Culp-Hill R, Nemkov T, Pei S, Khan N, Adane B, Ye H (2018). Inhibition of amino acid metabolism selectively targets human leukemia stem cells. Cancer Cell.

[12] Chapuis N, Poulain L, Birsen R, Tamburini J, Bouscary D (2019). Rationale for targeting deregulated metabolic pathways as a therapeutic strategy in acute myeloid leukemia. Front Oncol.

[13] Gandhi V, Ayres M, Halgren RG, Krett NL, Newman RA, Rosen ST (2001). 8-chloro-cAMP and 8-chloro-adenosine act by the same mechanism in multiple myeloma cells. Can Res.

[14] Stellrecht CM, Rodriguez CO, Ayres M, Gandhi V (2003). RNA-directed actions of 8-chloro-adenosine in multiple myeloma cells. Can Res.

[15] Stellrecht CM, Ayres M, Arya R, Gandhi V (2010). A unique RNA-directed nucleoside analog is cytotoxic to breast cancer cells and depletes cyclin E levels. Breast Cancer Res Treat.

[16] Balakrishnan K, Stellrecht CM, Genini D, Ayres M, Wierda WG, Keating MJ, Leoni LM, Gandhi V (2005). Cell death of bioenergetically compromised and transcriptionally challenged CLL lymphocytes by chlorinated ATP. Blood.

[17] Dennison JB, Balakrishnan K, Gandhi V (2009). Preclinical activity of 8-chloroadenosine with mantle cell lymphoma: roles of energy depletion and inhibition of DNA and RNA synthesis. Br J Haematol.

[18] Chen LS, Nowak BJ, Ayres ML, Krett NL, Rosen ST, Zhang S, Gandhi V (2009). Inhibition of ATP synthase by chlorinated adenosine analogue. Biochem Pharmacol.

[19] Stellrecht CM, Vangapandu HV, Le XF, Mao W, Shentu S (2014). ATP directed agent, 8-chloro-adenosine, induces AMP activated protein kinase activity, leading to autophagic cell death in breast cancer cells. J Hematol Oncol.

[20] Kearney AY, Fan YH, Giri U, Saigal B, Gandhi V, Heymach JV, Zurita AJ (2015). 8-Chloroadenosine sensitivity in renal cell carcinoma is associated with AMPK activation and mTOR pathway inhibition. PLoS ONE.

[21] Dennison JB, Shanmugam M, Ayres ML, Qian J, Krett NL, Medeiros LJ, Neelapu SS, Rosen ST, Gandhi V (2010). 8-Aminoadenosine inhibits Akt/mTOR and Erk signaling in mantle cell lymphoma. Blood.

[22] Buettner R, Nguyen LXT, Kumar B, Morales C, Liu C, Chen LS, Pemovska T, Synold TW, Palmer J, Thompson R (2019). 8-chloro-adenosine activity in FLT3-ITD acute myeloid leukemia. J Cell Physiol.

[23] Taylor CW, Yeoman LC (1992). Inhibition of colon tumor cell growth by 8-chloro-cAMP is dependent upon its conversion to 8-chloro-adenosine. Anticancer Drugs.

[24] Fang J, Shi Y, Zhang L (1995). Antitumor activities of 8-chloroadenosine in vivo and in vitro. Zhonghua Zhong Liu Za Zhi.

[25] Buettner R, Morales C, Caserta E, Troadec E, Gunes EG, Viola D, Khalife J, Li H, Keats JJ, Christofferson A (2019). Leflunomide regulates c-Myc expression in myeloma cells through PIM targeting. Blood Adv.

[26] Wang J, Li Y (2019). CD36 tango in cancer: signaling pathways and functions. Theranostics.

[27] Romero M, Sabate-Perez A, Francis VA, Castrillon-Rodriguez I, Diaz-Ramos A, Sanchez-Feutrie M, Duran X, Palacin M, Moreno-Navarrete JM, Gustafson B (2018). TP53INP2 regulates adiposity by activating beta-catenin through autophagy-dependent sequestration of GSK3beta. Nat Cell Biol.

[28] Wang YJ, Bian Y, Luo J, Lu M, Xiong Y, Guo SY, Yin HY, Lin X, Li Q, Chang CCY (2017). Cholesterol and fatty acids regulate cysteine ubiquitylation of ACAT2 through competitive oxidation. Nat Cell Biol.

[29] Ramos-Valdivia AC, van der Heijden R, Verpoorte R (1997). Isopentenyl diphosphate isomerase: a core enzyme in isoprenoid biosynthesis. A review of its biochemistry and function. Nat Prod Rep.

[30] DiNardo CD, Pratz KW, Letai A, Jonas BA, Wei AH, Thirman M, Arellano M, Frattini MG, Kantarjian H, Popovic R (2018). Safety and preliminary efficacy of venetoclax with decitabine or azacitidine in elderly patients with previously untreated acute myeloid leukaemia: a non-randomised, open-label, phase 1b study. Lancet Oncol.

[31] Nguyen LXT, Troadec E, Kalvala A, Kumar B, Hoang DH, Viola D, Zhang B, Nguyen DQ, Aldoss I, Ghoda L (2019). The Bcl-2 inhibitor venetoclax inhibits Nrf2 antioxidant pathway activation induced by hypomethylating agents in AML. J Cell Physiol.

