# Supplementary material for: Targeting the metabolic vulnerability of acute myeloid leukemia blasts with a combination of venetoclax and 8-chloro-adenosine
Source: J Hematol Oncol. 2021 Apr 26;14:70. doi: 10.1186/s13045-021-01076-4 (PMC8074444; doi:10.1186/s13045-021-01076-4)
Supplement: Supplementary file 1 — Additional file 1: Supplemental figures and tables. [file 13045_2021_1076_MOESM1_ESM.docx]

**Additional file**

**
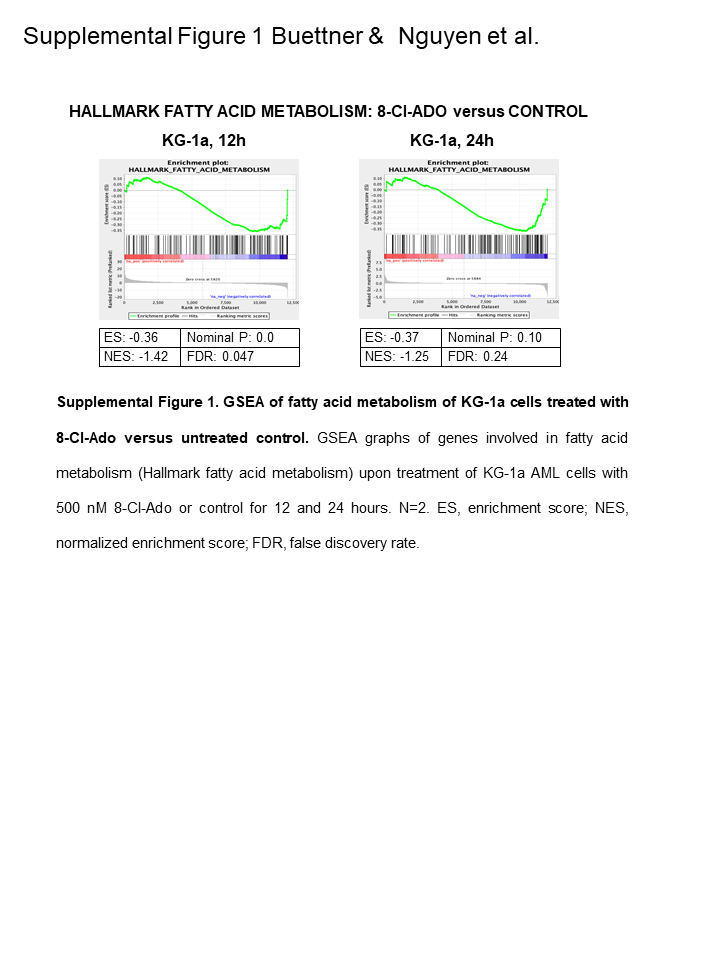
**

**Figure S1.** GSEA of fatty acid metabolism of KG-1a cells treated with 8-Cl-Ado versus untreated control. GSEA graphs of genes involved in fatty acid metabolism (Hallmark fatty acid metabolism) upon treatment of KG-1a AML cells with 500 nM 8-Cl-Ado or control for 12 and 24 hours. N=2. ES, enrichment score; NES, normalized enrichment score; FDR, false discovery rate.

**
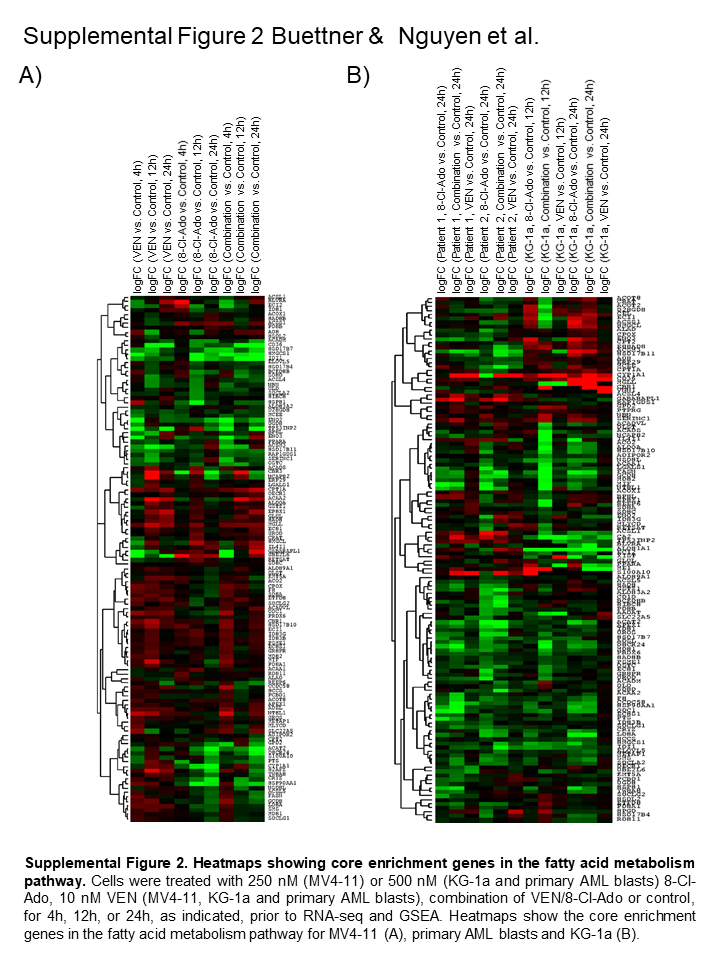
**

**Figure S2.** Heatmaps showing core enrichment genes in the fatty acid metabolism pathway. Cells were treated with 250 nM (MV4-11) or 500 nM (KG-1a and primary AML blasts) 8-Cl-Ado, 10 nM VEN (MV4-11, KG-1a and primary AML blasts), combination of VEN/8-Cl-Ado or control, for 4h, 12h, or 24h, as indicated, prior to RNA-seq and GSEA. Heatmaps show the core enrichment genes in the fatty acid metabolism pathway for MV4-11 (A), primary AML blasts and KG-1a (B).

**
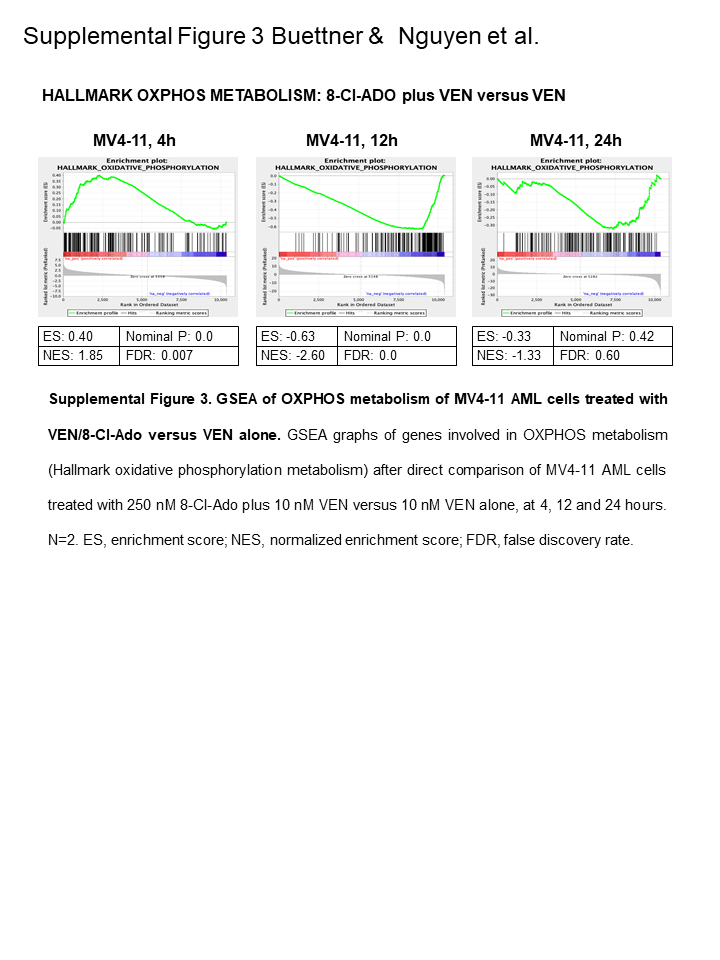
**

**Figure S3. GSEA of OXPHOS metabolism of MV4-11 AML cells treated with VEN/8-Cl-Ado versus VEN alone.** GSEA graphs of genes involved in OXPHOS metabolism (Hallmark oxidative phosphorylation metabolism) after direct comparison of MV4-11 AML cells treated with 250 nM 8-Cl-Ado plus 10 nM VEN versus 10 nM VEN alone, at 4, 12 and 24 hours. N=2. ES, enrichment score; NES, normalized enrichment score; FDR, false discovery rate.


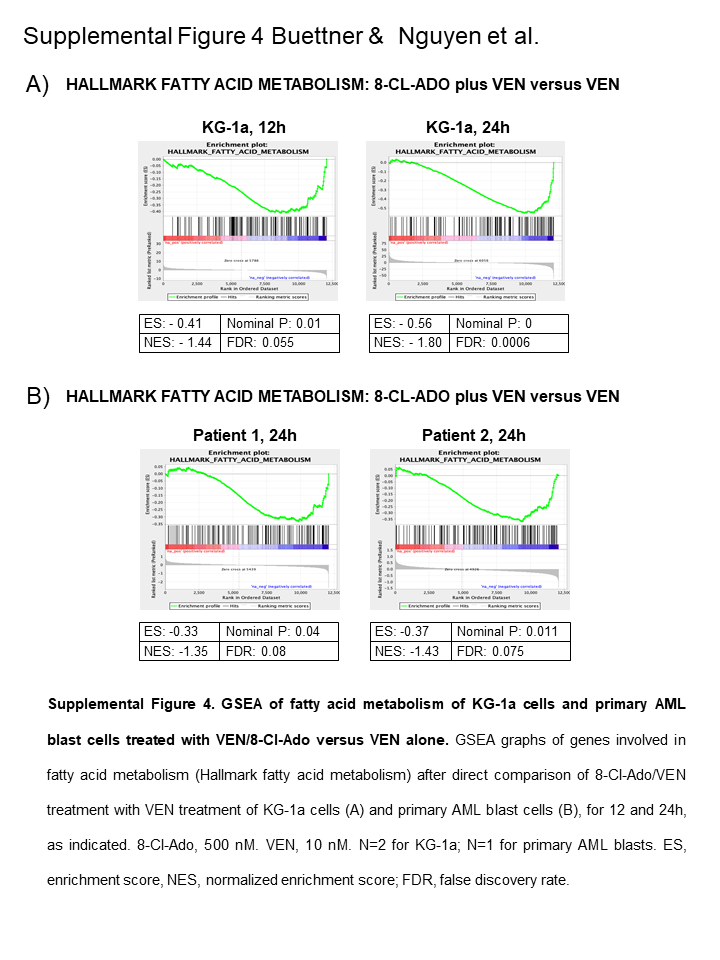


**Figure S4.** GSEA of fatty acid metabolism of KG-1a cells and primary AML blast cells treated with VEN/8-Cl-Ado versus VEN alone. GSEA graphs of genes involved in fatty acid metabolism (Hallmark fatty acid metabolism) after direct comparison of 8-Cl-Ado/VEN treatment with VEN treatment of KG-1a cells (A) and primary AML blast cells (B), for 12 and 24h, as indicated. 8-Cl-Ado, 500 nM. VEN, 10 nM. N=2 for KG-1a; N=1 for primary AML blasts. ES, enrichment score, NES, normalized enrichment score; FDR, false discovery rate.

**
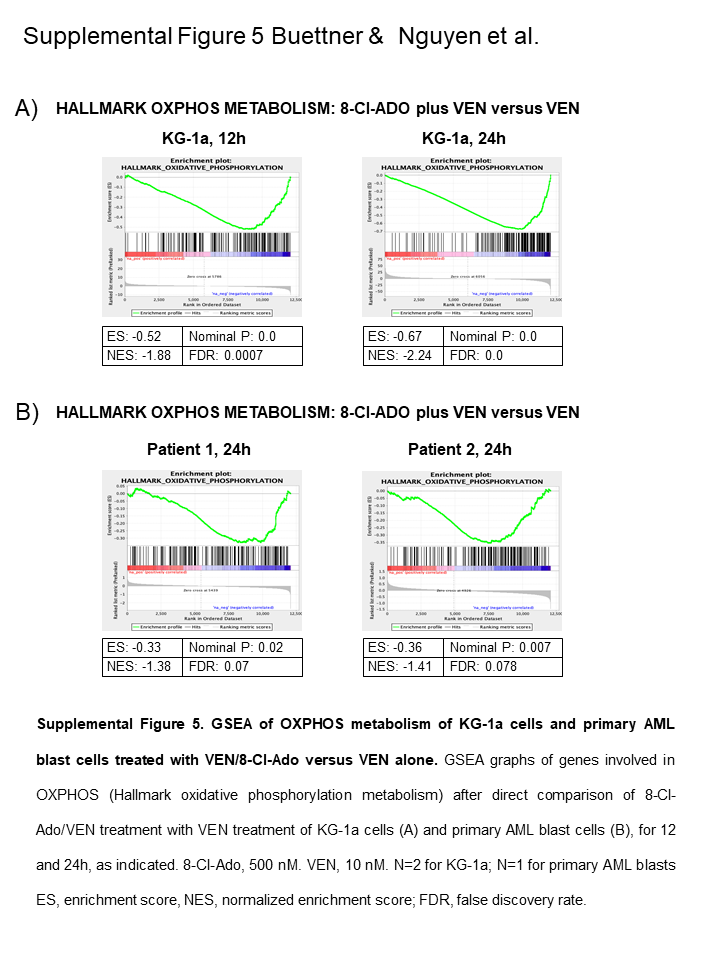
**

**Figure S5.** GSEA of OXPHOS metabolism of KG-1a cells and primary AML blast cells treated with VEN/8-Cl-Ado versus VEN alone. GSEA graphs of genes involved in OXPHOS (Hallmark oxidative phosphorylation metabolism) after direct comparison of 8-Cl-Ado/VEN treatment with VEN treatment of KG-1a cells (A) and primary AML blast cells (B), for 12 and 24h, as indicated. 8-Cl-Ado, 500 nM. VEN, 10 nM. N=2 for KG-1a; N=1 for primary AML blasts ES, enrichment score, NES, normalized enrichment score; FDR, false discovery rate.

**
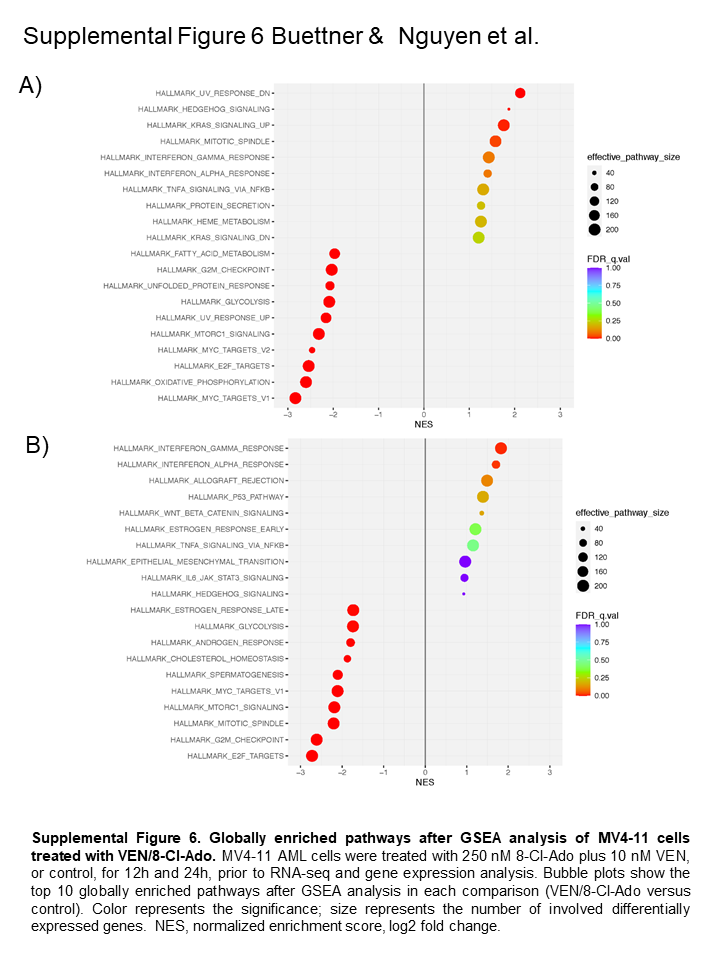
**

**Figure S6.** Globally enriched pathways after GSEA analysis of MV4-11 cells treated with VEN/8-Cl-Ado. MV4-11 AML cells were treated with 250 nM 8-Cl-Ado plus 10 nM VEN, or control, for 12h and 24h, prior to RNA-seq and gene expression analysis. Bubble plots show the top 10 globally enriched pathways after GSEA analysis in each comparison (VEN/8-Cl-Ado versus control). Color represents the significance; size represents the number of involved differentially expressed genes. NES, normalized enrichment score, log2 fold change.

**
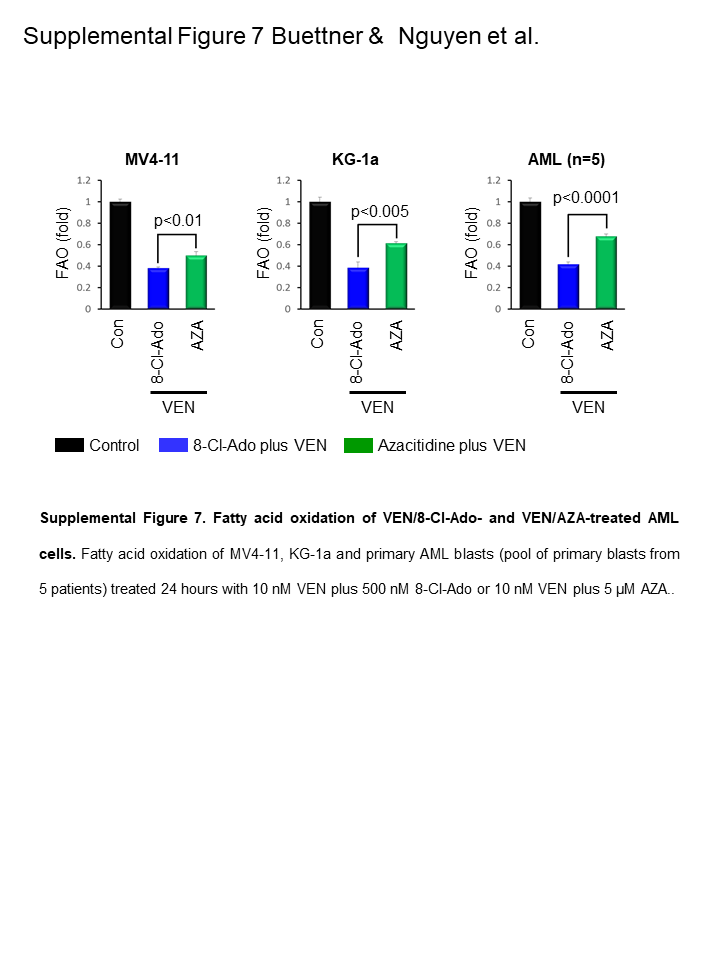
**

**Figure S7.** Fatty acid oxidation of VEN/8-Cl-Ado- and VEN/AZA-treated AML cells. Fatty acid oxidation of MV4-11, KG-1a and primary AML blasts (pool of primary blasts from 5 patients) treated 24 hours with 10 nM VEN plus 500 nM 8-Cl-Ado or 10 nM VEN plus 5 µM AZA.

**Supplemental Table 1**

**
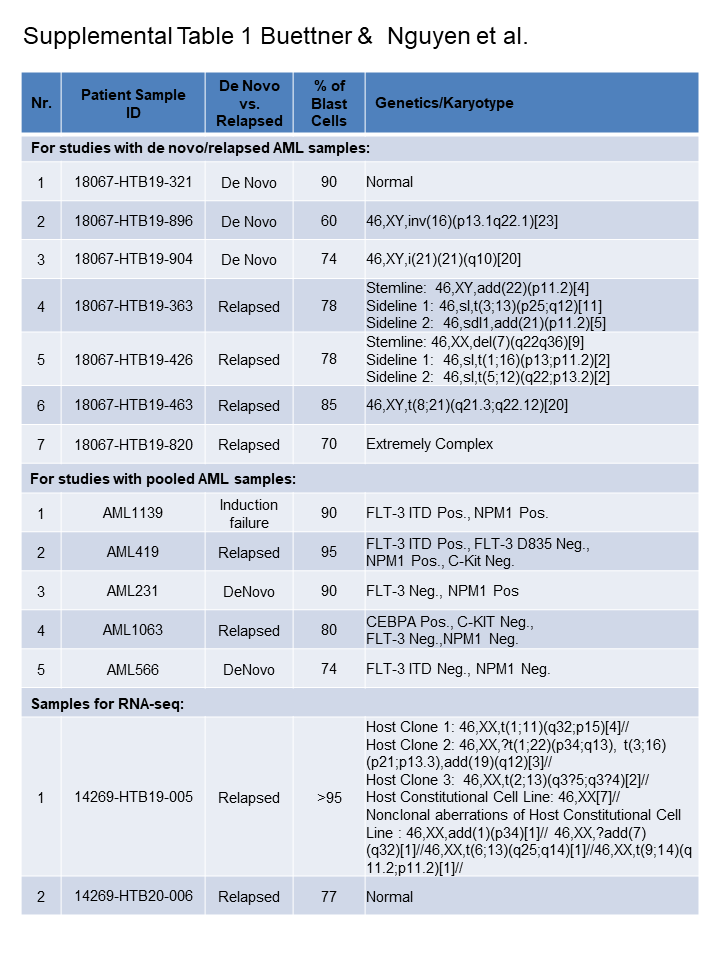
**

**Table S1.** Sample information for primary AML blast cells. Table containing patient number and patient sample ID number, *de novo* vs. relapsed patient classification, percent of blast cell count and genetics/karyotype for each of the primary AML blast samples used in the current studies, as known.

**
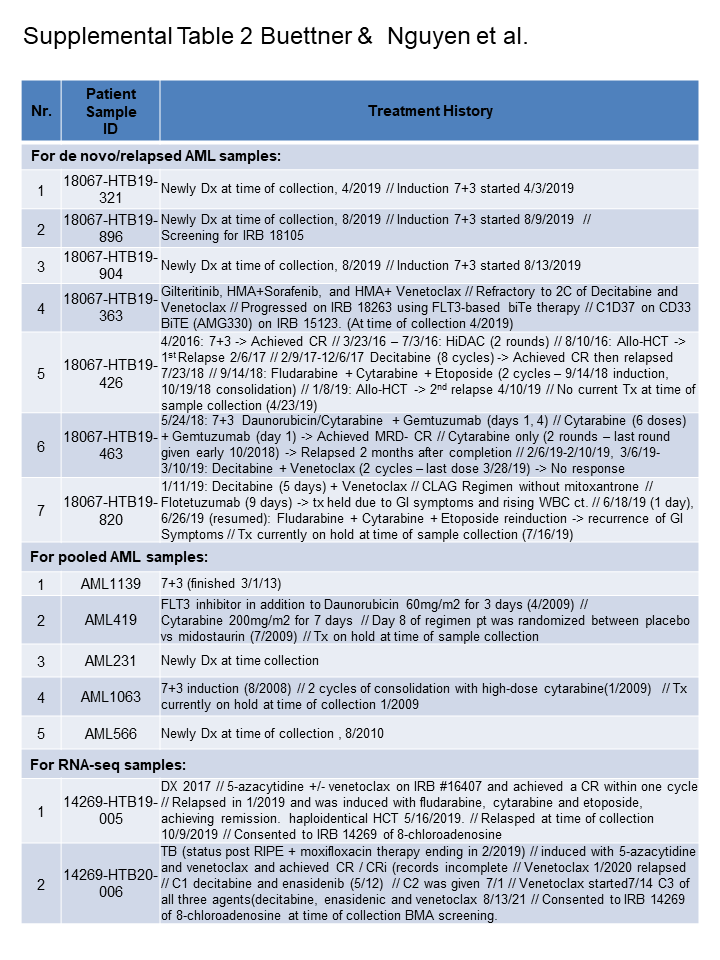
**

**Table S2.** Patient treatment history. For each of the primary AML blast samples used in the current studies, the corresponding associated patient treatment history is provided, if known.
